# SARS-CoV-2 Variants in COVID-19 Disease: A Focus on Disease Severity and Vaccine Immunity in Patients Admitted to the Emergency Department

**DOI:** 10.3390/jpm12122001

**Published:** 2022-12-02

**Authors:** Marta Fogolari, Maria Francesconi, Lucia De Florio, Marta Giovanetti, Roberta Veralli, Cecilia De Flora, Antonello Maruotti, Fabio Scarpa, Silvia Spoto, Federica Sambuco, Elisabetta Riva, Massimo Ciccozzi, Silvia Angeletti

**Affiliations:** 1 Operative Research Unit of Clinical Laboratory, Fondazione Policlinico Universitario Campus Bio-Medico, Via Alvaro del Portillo 200, 00128 Roma, Italy; 2 Unit of Clinical Laboratory Science, Università Campus Bio-Medico di Roma, Via Alvaro del Portillo 21, 00128 Roma, Italy; 3 Laboratório de Flavivírus, Instituto Oswaldo Cruz, Fundação Oswaldo Cruz, Rio de Janeiro 21040-900, Brazil; 4 Department of Science and Technology for Humans and the Environment, University of Campus Bio-Medico di Roma, 00128 Rome, Italy; 5 Unit of Virology, Università Campus Bio-Medico di Roma, Via Alvaro del Portillo 21, 00128 Roma, Italy; 6 Dipartimento di Scienze Economiche, Politiche e delle Lingue Moderne—Libera Università Maria Ss Assunta, 00193 Roma, Italy; 7 Department of Biomedical Sciences, University of Sassari, 07100 Sassari, Italy; 8 Diagnostic and Therapeutic Medicine Department, Policlinico Universitario Campus Bio-Medico, Via Alvaro del Portillo 200, 00128 Roma, Italy; 9 Emergency Department, Fondazione Policlinico Universitario Campus Bio-Medico, Via Alvaro del Portillo 200, 00128 Roma, Italy; 10 Unit of Medical Statistic and Molecular Epidemiology, Università Campus Bio-Medico di Roma, Via Alvaro del Portillo 21, 00128 Roma, Italy

**Keywords:** COVID-19, SARS-CoV-2, VOC, sequencing, vaccination, omicron

## Abstract

Tracking SARS-CoV-2 variants along with vaccinations are fundamental for severe COVID-19 disease prevention. A study was performed that focused on 43 patients with the SARS-CoV-2 infection who were admitted to the Emergency Department. RT-PCR–positive nasopharyngeal samples were sequenced using the MiSeq II system for variant detection. The main reason for Emergency Department admission was COVID-19 (67%), followed by other causes (33%); 51% patients were unvaccinated or vaccinated with a single dose and 49% had completed the vaccination course with two or three doses. Among the vaccinated group, 38% were admitted for COVID-19, versus 94.5% of the unvaccinated group. After admission, 50% of the vaccinated group and 36% of the unvaccinated group were discharged and allowed to go home, and 80% of the unvaccinated had no major comorbidities; 63% needed hospital admission and 5% required a stay in the Intensive Care Unit. Of these, 37% were vaccinated with 3 doses, 11% with two doses, 4% with a single dose, and 48% were unvaccinated. The 70% of the vaccinated patients who were admitted to hospital presented major comorbidities versus 38% of the unvaccinated group. Two unvaccinated patients that needed intensive care had relevant comorbidities and died. Genome sequencing showed the circulation of three omicron and two pure sub-lineages of omicron, including 22 BA.1, 12 BA.1.1, and 7 BA.2. Data showed the SARS-CoV-2 national and international migration patterns and how vaccination was useful for severe COVID-19 disease prevention.

## 1. Introduction

In the last year, the need to understand the biological and clinical significance of the emerging variants of SARS-CoV-2 was dominant in the scientific field. These variants showed an increase in the transmissibility of the virus, in the rate of re-infection, and a decrease in the degree of protection evoked by neutralizing antibodies and vaccination [1].

Viral variants, which represent an evolutionary advantage for the virus, allow it to acquire better fitness, and consequently, promote its spread through the population.

Since December 2020, five different lineages of the virus have been identified, which the World Health Organization (WHO), the US Centers for Disease Control and Prevention (CDC), and the COVID-19 Genomics UK Consortium (COG-UK), have called Variants of Concern (VOC). The WHO used a nomenclature based on the Greek alphabet by naming the emerging VOCs from May 2021 as Alpha, Beta, Gamma, Delta, and Omicron [2,3,4]. 

Next to this nomenclature, another has been added, which has been assigned by the Phylogenetic Assignment of Named Global Outbreak (PANGO) (https://cov-lineages.org/) this is the most accredited epidemiological surveillance system, and it is based on the use of an alphabetic prefix followed by a suffix containing up to three numbers separated by periods. According to this, the VOCs are called: Alpha (B.1.1.7), Beta (B.1.351), Gamma (P.1), Delta (B.1.617.2), and Omicron (2021.1) [5].

Moreover, with the aim of sharing as much data on emerging variants as possible on an international level, the genomic sharing platform, GISAID (https://gisaid.org/) was introduced to provide quick and open access to data on epidemic and pandemic viruses, including SARS-CoV-2.

In this context, capillary epidemiological surveillance was implemented to monitor the circulation of VOCs.

Currently, one of the ways to prevent severe SARS-CoV-2 infection is vaccination. This preventive measure should be extended to the global population to the highest degree possible, even in countries where vaccine accessibility is low and the risk of infection is high. Equality in access to vaccination would mean reducing the spread and replication of the virus, thus lowering the possibility of the emergence of new variants whose pathogenicities are not known [6].

In the absence of global immunization, the evolution of the virus boosts the chance that there will be an emergence of new variants.

In this study, the circulation of VOCs among patients admitted to the Emergency Department (ED) at the Fondazione Policlinico Universitario Campus Bio-Medico, in Rome, Italy, between January and March 2022 was evaluated in conjunction with vaccination status and reasons for ED admission; a correlation analysis of the patients’ outcomes was performed. This analysis could provide new information concerning the evolution of COVID-19 following, or without, vaccination and it may contribute to the collection of scientific and clinical data on this matter.

## 2. Materials and Methods

### 2.1. Patients

In this study, 43 patients who were admitted to the ED at the Fondazione Policlinico Universitario Campus Bio-Medico, in Rome, Italy, between 22 January and 7 March 2022, were included. They tested positive in Real Time (RT) after a Polymerase Chain Reaction (PCR) test for the detection of SARS-CoV-2 using nasopharyngeal swabs. 

### 2.2. Viral RNA Extraction and RT-PCR Reaction

The Microlab Nimbus (Hamilton Italia Srl, Agrate Brianza, Italy) 72 batch sample loading system (which is a compact, automated, multi-channel, fast, and flexible, instrument) was used to perform molecular tests for the identification of SARS-CoV-2 with nasopharyngeal swabs. This was achieved by automatically extracting RNA and preparing RT-PCR reaction plates (Allplex™ 2019-nCoV Assay (Seegene Inc.; Seoul, Republic of Korea). Amplification was performed with a Real Time-PCR amplifier on CFX96 ™ Dx (Bio-Rad Laboratories, Inc., Segrate, Milano, Italy) and an analysis of the results was carried out on dedicated Seegene Viewer software. The method foresees that the detection of the target gene takes place when the Cycle Threshold (Ct) is lower than 40. The test detects the E, N, RdRP/S genes. The analytical sensitivity declared by the kit is equal to 4.167 copies/μL for gene E, 1250 copies/μL for the RdRP gene, and 4167 copies/μL for the N gene, respectively. Regarding the analytical specificity, the analysis performed in silico did not show cross-reactivity with the 65 potential respiratory pathogens tested (Allplex™ 2019-nCoV Assay-Seegene). All the samples sequenced were selected and identified exclusively in accordance with these criteria.

### 2.3. Whole Genome Sequencing for VOCs Identification

The RT-PCR–positive samples were submitted for genomic sequencing using the MiSeq II system (Illumina, Milano, Italy), in accordance with the manufacturer’s instructions. Consensus sequences were generated by de novo assembling using the iVar with the default setting [7]. A lineage assignment was performed using the Pangolin lineage classification software tool [5].

### 2.4. Statistical Analysis

Percentages, where applicable, were compared with the ꭕ^2^ test in order to give proportions. A *p* value < 0.05 was considered significant.

## 3. Results

### 3.1. Study Population

The study consisted of 25 males and 18 females with a mean age of 65 ± 17.0 years (mean ± sd). Among the selected patients, 10 (23%) had no comorbidities, whereas 33 (77%) had two or more comorbidities. The presence of comorbidities in patients admitted to the ED was statistically relevant (*p* = 0.0020). The most frequently encountered comorbidities were systemic arterial hypertension, cardiovascular disease, obesity, dyslipidemia, type 2 diabetes mellitus, and neoplastic disease which occurs during anti-cancer treatment.

We analyzed the main reasons for ED admission; 67% of patients were admitted for symptoms due to COVID-19, and among these, fever, sore throat, and cold were detected in 23% of cases, followed by dyspnea (21%) and abdominal or chest pain (23%).

**Figure 1:**
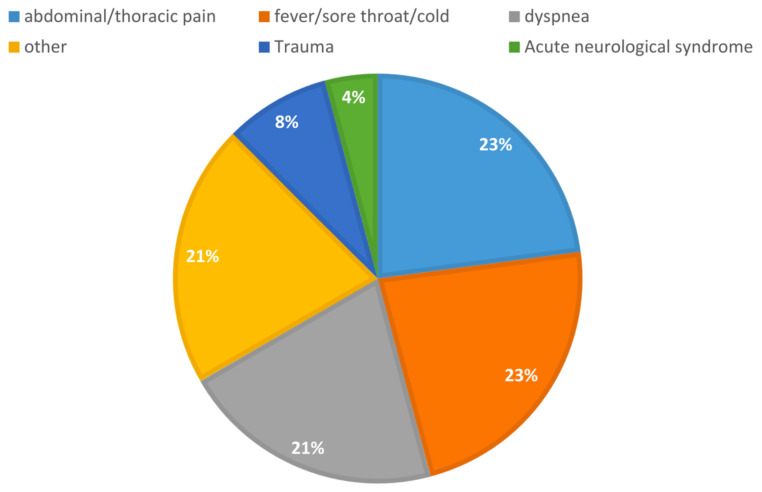
Causes for Emergency Department admission in the study population.

The remaining 33% were admitted for other causes: 8% for trauma, 21% of cases presented other symptoms that were not attributable to COVID-19, and 4% had acute neurological syndrome, as reported in Figure 1. The proportion of patients admitted to the ED for COVID-19 related symptoms was statistically relevant (*p* = 0.0017).

At ED admission, patients had high median C-reactive Protein (CRP) levels (3.84 mg/dL, IQR 0.86–11.67—(normal values: ≤0.5 mg/dL), median values of leukocytes at 8.05 × 10^3^/μL, IQR 6.69–10.64 (normal values: 4–11 × 10^3^/μL) with a median neutrophil of 5.63 × 10^3^/μL, IQR 4.29–7.95 (normal values: 2–7 × 10^3^/μL), median lymphocyte values of 1.05 × 10^3^/μL, IQR 0.66–1.51 (normal values: 1–3 × 10^3^/μL), and a median high D-dimer of 1490 ng/mL, IQR 677–3380 (normal values:0–500 ng/mL).

Of the 43 patients who were included in the study, 22 (51%) patients were unvaccinated for SARS-CoV-2 or vaccinated with a single dose, and 21 (49%) had completed the vaccination course with two or three doses. Patients received mRNA vaccines from Comirnaty (BNT162b2) (Pfizer/BioNTech, Mainz, Germany/New York, NY, USA) or Spikewax (mRNA 1273) (Moderna TX, Cambridge, MA, USA). Of the latter 21 patients that were vaccinated, as many as 13 (52%) were admitted for trauma (fracture or accident) or for other symptoms that were not related to COVID-19 (renal colic, acute diverticulitis), whereas 8 (38%) were admitted as they presented a COVID-19-related symptomatology.

Of the 22 unvaccinated patients, only one patient (4.5%) was admitted for symptoms that could not be attributed to COVID-19 (dehydration and acute kidney failure in a patient with ileostomy), whereas the other 21 (94.5%) were admitted for COVID-19. Thus, 38% of the vaccinated presented a COVID-19 symptomatology, whereas among the unvaccinated, as many as 94.5% were admitted for presenting a COVID-19 symptomatology. The proportion of unvaccinated patients that were admitted to the ED for a COVID-19 symptomatology was statistically relevant (*p* < 0.0001).

After admission to the ED, 14/43 (32.5%) patients were discharged home. The proportion of patients needing hospital admission was statistically relevant (*p* = 0.013). Among the discharged patients, 7/14 (50%) were patients who were vaccinated with three doses of the vaccine, 1/14 (7%) were vaccinated with a single dose but had suffered from a previous SARS-CoV-2 infection, 1/14 (7%) were vaccinated with a single dose of the vaccine, and 5/14 (36%) were unvaccinated, as reported in Table 1. The proportion of vaccinated patients that was discharged home was statistically significant (*p* = 0.027).

Among the unvaccinated patients, only 1/5 (20%) had a comorbidity (diabetes); the remaining 4/5 (80%) had no major comorbidities, as reported in Table 2. The patients who had received a single dose of vaccine presented no comorbidities, whereas the patients vaccinated with a single dose, and who had suffered a prior SARS-CoV-2 infection, were affected by hypertension. Among patients who had received three doses of the vaccine 1/7 (14%) had two comorbidities (diabetes and hypertension) and 1/7 (14%) reported a prior cerebral hemorrhage, resulting in hemiparesis (Table 2). 

After admission to the ED, 27/43 (63%) needed hospital admission and 2/43 (5%) required a stay in the Intensive Care Unit. Of these, 10/27 (37%) were patients that were vaccinated with three doses of the vaccine, 3/27 (11%) were vaccinated with two doses or with a single dose, but who had suffered from a previous SARS-CoV-2 infection, 1/27 (4%) were vaccinated with a single dose of the vaccine, and 13/27 (48%) were unvaccinated, as reported in Table 1. 

The correlation between patients’ comorbidities, their vaccination status, and their destiny after being admitted to the ED (either discharged home or admitted to hospital) was analyzed, as reported in Table 2. Among the unvaccinated patients who were discharged home, 1/5 (20%) were affected by diabetes, whereas among the patients who were discharged home and who had three doses of the vaccine, 1/7 (14%) had both diabetes and hypertension and 1/7 (14%) presented hemiparesis due to prior cerebral hemorrhage. Furthermore, one vaccinated patient with one dose plus infection was discharged home and was affected by hypertension. 

Regarding hospital admission, vaccinated patients who had three doses of the vaccine presented comorbidities in 7/10 (70%) of cases; they were affected by neoplastic disease in 3/10 (30%) of cases, diabetes plus hypertension and COPD in 1/10 (10%) of cases, cardiomiopathy plus hypertension in 1/10 (10%) of cases, and COPD in 2/10 (20%) of cases (Table 2). Among the unvaccinated patients needing hospital admission, comorbidities were present in 5/13 (38%) of cases; 1/13 (8%) had neoplastic disease, 3/13 (23%) had diabetes plus hypertension, and 1/13 (8%) had diabetes plus heart and renal failure (Table 2). One patient who had taken a single dose of the vaccine was affected by chronic heart failure, and three patients who had taken two doses of the vaccine, or who had taken one dose and had previously suffered from the SARS-CoV-2 infection, had hypertension, diabetes, and neoplastic disease, and therefore, they required hospital admission (Table 2). Finally, after being admitted to the ED two patients needed ICU admission, and they presented significant comorbidities; one suffered heart failure (50%) and the other suffered from diabetes plus heart and renal failure (50%). Neither patient survived, as shown in Table 2.

### 3.2. SARSCoV-2 Identification and Sample Sequencing

For all the patients who tested positive for SARS-CoV-2 after taking the RT-PCR test using a nasopharyngeal swab, the RT-PCR cycle cutoffs (Cts) averaged 23.33 (with a range: 15.0 to 36.0).

**Figure 2:**
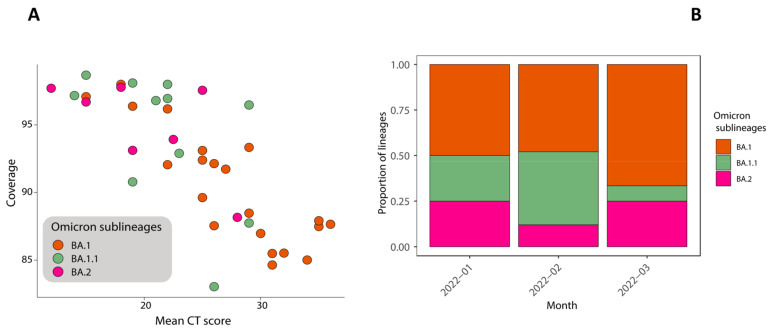
Genome coverage plotted against the mean threshold value of the RT-PCR cycle (CT score); colors represent different omicron sub-lineages (panel **A**). Frequency and distribution of the SARS-CoV-2 omicron sub-lineages from the whole-genome sequences generated within the study (panel **B**).

Following collection and analysis of the RT-PCR tests, we genotyped 43 samples; these contained sufficient viral genetic material (≥2 ng/µL) for the preparation of the library. Sequences had a mean genome coverage of 92.51% (range: 84.64 to 98.68) and the mean genome coverage was typically higher for samples with low Ct values, as shown in Figure 2A. 

For the analyzed samples, we can observe that from January to March 2022, there three omicron sub-lineages were in circulation; in particular, we identified two genomes that can be classified as pure omicron, which were 22 BA.1, 12 BA.1.1, and 7 BA.2. The frequency and distribution of the SARS-CoV-2 omicron sublines generated within the study is shown in Figure 2B. Starting from March 2022, we can observe that BA.2 increases the prevalence of the disease slightly while both BA.1 and BA.1.1 were still present in the study population.

For the data reported so far, there was no difference in disease severity that was associated with the two omicron sub-lineages. We can make a comparison with the data reported in the latest Istituto Superiore di Sanità (ISS) REPORT no. 21 of 1 July 2022 (data updated to June 2022) (https://www.epicentro.iss.it/coronavirus/pdf/sars-cov-2-monitoraggio-varianti-rapporti-periodici-1-luglio-2022.pdf). After comparing data, we can see that in January 2022, BA.1 and BA.1.1 were still prevalent. Although still rare at a national level, BA.2 was also present in our study population. In accordance with the ISS report, in the last days of February and March, there was a significant increase in the proportion of sequences that were attributable to the BA.2 sub-lineage.

## 4. Discussion

The COVID-19 pandemic represented a sudden event that shocked the world and captured the attention of all scientists who were trying to investigate the biology of this new virus, particularly its ability to adapt to humans. The aim was to find an increasing number of effective strategies to limit its spread and pathogenicity. This goal could be achieved through the introduction of new drugs, producing an effective vaccine in very short time, and by implementing rapid epidemiological surveillance for the prompt detection of new viral variants. In this context, the quick identification of emerging VOCs, their genomic sequencing, and the application of specific bio-informatic analyses, allowed the early detection of viral lineages with a higher frequency rate, thus enabling the implementation of even more effective prevention strategies. As recently suggested, once a variant of interest is defined as a VOC, to reduce its spread, different approaches are used, including effective genetic surveillance, which provides information concerning virus evolutionary changes from the start of the pandemic, and it assists with public health measures [8,9].

The omicron variant Is now the most predominant strain in our country (prevalence estimated at national level equal to 99.9%, data source https://www.iss.it, accessed on 1 July 2022. Based on the latest available evidence, the WHO Technical Advisory Group on SARS-CoV-2 Virus Evolution (TAG-VE) suggests that the omicron variant should continue to be closely monitored in order to accumulate evidence of its transmissibility (Tracking SARS-CoV-2 variants (https://www.who.int, accessed on 1 July 2022)).

The emergence of new viral variants requires limiting the spread of infection by promoting vaccination, improving surveillance by genomic sequencing of SARS-CoV2 variants, and global data sharing [6,9].

Based on recent data, omicron was found to be more transmissible than the delta variant; however, patients did not show unusual symptoms, with some of them presenting as asymptomatic. The WHO and CDC alerted healthcare systems so that they could plan the most efficient way to contain the spread of the variant in the population from the moment of its introduction [10,11].

Regarding vaccine efficacy against the omicron variant and long-term vaccine protection, data are being collected; however, it has been suggested that it is likely that vaccines can guarantee a certain degree of protection, and above all, it can help avoid the establishment of severe disease, as suggested by data reported from Israel and the UK, who recommended booster doses [12,13,14,15].

Our study seems to suggest that completing the vaccination course is essential to the prevention of severe illness, since most vaccinated patients were admitted to the ED for reasons other than COVID-19, whereas almost all, except one, of the unvaccinated patients were admitted for presenting a COVID-19 disease symptomatology. Furthermore, 50% of vaccinated patients who had taken three doses were discharged home, versus 38% of the unvaccinated patients admitted to the ED. Among the patients discharged home, the comorbidities were similarly distributed between the two groups of unvaccinated and vaccinated patients; however, if we analyze the comorbidities among the hospitalized patients, we can observe that the comorbidities were more prevalent in the vaccinated group than in the unvaccinated group (70% versus 38%). It should also be noted that the two patients who needed hospitalization in intensive care were both unvaccinated, they had major comorbidities (diabetes, kidney, and heart failure), and they both died. These data are in accordance with other recent studies confirming that patients who were vaccinated were significantly more protected from severe COVID-19 compared with the unvaccinated group [16,17,18,19,20].

Furthermore, as reported by other authors, a decreased vaccine efficacy in patients with comorbidities was observed. Antibody positivity was lower in patients receiving organ transplantation, or it was affected by hematological malignancies, such as obesity and smoking habits. Moreover, the prominence of the disease was significantly correlated with age and the female sex [21,22,23].

To date, a large proportion of the population belonging to low-income countries has not yet been vaccinated; therefore, these people are at greater risk for severe disease development that needs hospitalization, and this promotes the potential emergence of new mutations during virus evolution. For these reasons, it is essential to assure adequate vaccine coverage around the world, and data from our study focuses precisely on this issue. Moreover, we know that some variants have increased transmissibility [24], and thus, the importance of efficient public health measures and vaccination programs has to be increases. The global response must be both timely and science-based. Some priorities have to take into account the fact that we must evaluate existing vaccines for efficacy against variants over time, and in this sense, it is important to assess the effectiveness of new vaccines or modified vaccines against new circulating variants. Finally, it is important to reduce the risk of additional variants emerging over time by promoting public health measures (e.g., masking, social distancing, and vaccination) to reduce viral transmission and drive the evolution of variants in such a manner that humans can adapt to it.

### Limitation of the Study

Some limitations of the study can be summarized here, as the relatively small sample size of the study and the lack of data regarding the vaccination response by the antibodies alter the measurements. Further studies could be performed to enlarge the sample size and investigate serum antibodies titers after vaccination. In this sense, new insights regarding vaccination and immunological response could enrich data from the present study with more information that is useful for a better comprehension of COVID-19, thus improving patient management and outcomes.

## 5. Conclusions

Our findings reinforce the fact that virus migration has generally followed patterns of national and international human mobility, thus illustrating how vaccination can be a valid tool to prevent severe COVID-19, even when the vaccinated individual becomes infected. We have learned that facing a pandemic requires one health approach that integrates different plans of action following a multidisciplinary strategy, among which, vaccination and completing the vaccination course represent very useful tools which guarantee the population the most effective protection. It is also important to avoid the use of treatments with uncertain benefits that could drive the evolution of variants.

## Figures and Tables

**Table 1:** Vaccination status and patient management after Emergency Department admission.

Vaccination Status	Discharge Home *n*.(%)	Hospital Admission *n*.(%)	Intensive Care Unit Admission *n*. (%)	30-Day Mortality *n*. (%)
No dose	5 (36)	13 (48)	2 * (100)	2 * (100)
One dose	1 (7)	1 (4)	0	0
Two doses or one dose plus infection	1 (7)	3 (11)	0	0
Three doses	7 (50)	10 (37)	0	0
Total	14 (100)	27 (100)	2 (100)	2 (100)

* not-surviving patients.

**Table 2:** Vaccination status and patient management shown in conjunction with the patients’ comorbidities.

**Vaccination Status**	**Patients Discharged Home *n*. (%)**	**Patients with Comorbidities** **(*n*., %)**
No dose	5 (36)	Diabetes (1, 20)
One dose	1 (7)	None
Two doses or one dose plus infection	1 (7)	Hypertension (1, 100)
Three doses	7 (50)	-Diabetes + Hypertension (1, 14) -Prior cerebral hemorrhage resulting in hemiparesis (1, 14)
Total	14 (100)	4 (28)
**Vaccination status**	**Patients admitted to** **hospital** ***n*. (%)**	**Patients with comorbidities** **(*n*., %)**
No dose	13	-Neoplastic disease (1, 8) -Diabetes + Hypertension (3, 23) -Diabetes + Heart and Renal Failure (1, 8)
One dose	1	Heart Failure (1, 100)
Two doses or one dose plus infection	3	-Neoplastic disease (1, 33) -Diabetes (1, 33) -Hypertension (1, 33)
3 dose	10	-Neoplastic disease (3, 30) -Diabetes + Hypertension + COPD (1, 10) -Cardiomiopathy + Hypertension (1, 10) -COPD (2, 20)
Total	27 (100)	7 (70)
**Vaccination status**	**Patients admitted to Intensive Care Unit *n*. (%)**	**Patients with comorbidities** **(*n*., %)**
None	2 (100)	-Heart Failure (1, 50) -Diabetes + Heart and Renal Failure (1, 50)
Total	2 (100) *	2 (100) *

* non-surviving patients.

## Data Availability

Newly generated SARS-CoV-2 sequences have been deposited in GISAID under accession numbers: EPI_ISL_13566018, EPI_ISL_13566019, EPI_ISL_11148626, EPI_ISL_11149332, EPI_ISL_11149367, EPI_ISL_11149332, EPI_ISL_11149372, EPI_ISL_11149532, EPI_ISL_11149534, EPI_ISL_11149538, EPI_ISL_11149540, EPI_ISL_11149560, EPI_ISL_11149563, EPI_ISL_11149613, EPI_ISL_11149616, EPI_ISL_11149638, EPI_ISL_11149642, EPI_ISL_11149738, EPI_ISL_11149740, EPI_ISL_11151072, EPI_ISL_11151276, EPI_ISL_11222945, EPI_ISL_12030819, EPI_ISL_12030821, EPI_ISL_12030822, EPI_ISL_12031068, EPI_ISL_12031318, EPI_ISL_12031527, EPI_ISL_13566020, EPI_ISL_13566021, EPI_ISL_13566022, EPI_ISL_13566023, EPI_ISL_13566025, EPI_ISL_13566026, EPI_ISL_13566027, EPI_ISL_13566028, EPI_ISL_13566029, EPI_ISL_13566030, EPI_ISL_13566031, EPI_ISL_13566032, EPI_ISL_13566033, EPI_ISL_13566035, EPI_ISL_13566036.

## References

[1] Tao K., Tzou P.L., Nouhin J., Gupta R.K., de Oliveira T., Kosakovsky Pond S.L., Fera D., Shafer R.W. (2021). The biological and clinical significance of emerging SARS-CoV-2 variants. Nat. Rev. Genet..

[2] WHO (2021). SARS-CoV-2 Variants of Concern and Variants of Interest.

[3] CDC (2021). SARS-CoV-2 Variant Classifications and Definitions.

[4] The COVID-19 Genomics UK (COG-UK) Consortium (2020). An integrated national scale SARS-CoV-2 genomic surveillance network. Lancet Microbe.

[5] Rambaut A., Holmes E.C., O’Toole A., Hill V., McCrone J.T., Ruis C., du Plessis L., Pybus O.G. (2020). A dynamic nomenclature proposal for SARS-CoV-2 lineages to assist genomic epidemiology. Nat. Microbiol..

[6] Fontanet A., Autran B., Lina B., Kieny M.P., Karim S.S.A., Sridhar D. (2021). SARS-CoV-2 variants and ending the COVID-19 pandemic. Lancet.

[7] Grubaugh N.D., Gangavarapu K., Quick J., Matteson N.L., De Jesus J.G., Main B.G., Tan M.L., Paul L.M., Brackney D.E., Grewal S. (2019). An amplicon-based sequencing framework for accurately measuring intrahost virus diversity using PrimalSeq and iVar. Genome Biol..

[8] Otto S.P., Day T., Arino J., Colijn C., Dushoff J., Li M., Mechai S., Van Domselaar G., Wu J., Earn D.J.D. (2021). The origins and potential future of SARS-CoV-2 variants of concern in the evolving COVID-19 pandemic. Curr. Biol..

[9] He X., Hong W., Pan X., Lu G., Wei X. (2021). SARS-CoV-2 Omicron variant: Characteristics and prevention. Med. Commun..

[10] Daria S., Bhuiyan M.A., Islam M.R. (2022). Detection of highly muted coronavirus variant Omicron (B.1.1.529) is triggering the alarm for South Asian countries: Associated risk factors and preventive actions. J. Med. Virol..

[11] Saxena S.K., Kumar S., Ansari S., Paweska J.T., Maurya V.K., Tripathi A.K., Abdel-Moneim A.S. (2022). Characterization of the novel SARS-CoV-2 Omicron (B.1.1.529) Variant of Concern and its global perspective. J. Med. Virol..

[12] Patalon T., Gazit S., Pitzer V.E., Prunas O., Warren J.L., Weinberger D.M. (2022). Odds of testing positive for SARS-CoV-2 following receipt of 3 vs. 2 doses of the BNT162b2 mRNA vaccine. JAMA Intern. Med..

[13] Bar-On Y.M., Goldberg Y., Mandel M., Bodenheimer O., Freedman L., Kalkstein N., Mizrahi B., Alroy-Preis S., Ash N., Milo R. (2021). Protection of BNT162b2 vaccine booster against COVID-19 in Israel. N. Engl. J. Med..

[14] Andrews N., Stowe J., Kirsebom F., Gower C., Ramsay M., Bernal J.L. (2022). Effectiveness of BNT162b2 (Comirnaty, Pfizer-BioNTech) COVID-19 booster vaccine against COVID-19 related symptoms in England: Test negative case-control study. Nat. Med..

[15] Centers for Disease Control and Prevention (2021). CDC Expands COVID-19 Booster Recommendations. https://www.cdc.gov/media/releases/2021/s1129-booster-recommendations.html.

[16] Wichaidit M., Nopsopon T., Sunan K., Phutrakool P., Ruchikachorn P., Wanvarie D., Pratanwanich P.N., Cheewaruangroj N., Punyabukkana P., Pongpirul K. (2023). Breakthrough infections, hospital admissions, and mortality after major COVID-19 vaccination profiles: A prospective cohort study. Lancet Reg. Health Southeast Asia.

[17] Babouee F.B., Güsewell S., Egger T., Leal O., Brucher A., Lemmenmeier E., Meier Kleeb D., Möller J.C., Rieder P., Rütti M. (2022). Risk and symptoms of COVID-19 in health professionals according to baseline immune status and booster vaccination during the Delta and Omicron waves in Switzerland-A multicentre cohort study. PLoS Med..

[18] Kshirsagar M., Nasir M., Mukherjee S., Becker N., Dodhia R., Weeks W.B., Ferres J.L., Richardson B. (2022). The Risk of Hospitalization and Mortality After Breakthrough SARS-CoV-2 Infection by Vaccine Type: Observational Study of Medical Claims Data. JMIR Public Health Surveill..

[19] Stalman E.W., Wieske L., van Dam K.P.J., Kummer L.Y., van Kempen Z.L.E., Killestein J., Volkers A.G., Tas S.W., Boekel L., Wolbink G.J. (2022). T2B immunity against SARS-CoV-2 study group. Breakthrough infections with the SARS-CoV-2 omicron (B.1.1.529) variant in patients with immune-mediated inflammatory diseases. Ann. Rheum. Dis..

[20] Bajči M.P., Lendak D.F., Ristić M., Drljača M.M., Brkić S., Turkulov V., Petrović V. (2022). COVID-19 Breakthrough Infections among Patients Aged ≥65 Years in Serbia: Morbidity and Mortality Overview. Vaccines.

[21] Ward H., Whitaker M., Flower B., Tang S.N., Atchison C., Darzi A., Donnelly C.A., Cann A., Diggle P.J., Ashby D. (2022). Population antibody responses following COVID-19 vaccination in 212,102 individuals. Nat. Commun..

[22] Guven D.C., Sahin T.K., Kilickap S., Uckun F.M. (2021). Antibody Responses to COVID-19 Vaccination in Cancer: A Systematic Review. Front. Oncol..

[23] Sakuraba A., Luna A., Micic D. (2022). A Systematic Review and Meta-Analysis of Serologic Response following Coronavirus Disease 2019 (COVID-19) Vaccination in Solid Organ Transplant Recipients. Viruses.

[24] Pascarella S., Ciccozzi M., Bianchi M., Benvenuto D., Cauda R., Cassone A. (2022). The value of electrostatic potentials of the spike receptor binding and N-terminal domains in addressing transmissibility and infectivity of SARS-CoV-2 variants of concern. J. Infect..

